# Outcomes of conservative antibiotic therapy in pediatric peritonsillar abscesses: a retrospective observational study

**DOI:** 10.1007/s00431-025-06463-4

**Published:** 2025-09-18

**Authors:** Francesca Hoegger, Sandra Andrea Asner, Sophie Fries, Jean-Yves Pauchard, Pierre Alex Crisinel

**Affiliations:** 1 Service of Pediatrics, Department Woman-Mother-Child, Lausanne University Hospital and University of Lausanne, Lausanne, Switzerland; 2 Unit of Pediatric Infectious Diseases and Vaccinology, Service of Pediatrics, Department Woman-Mother-Child, Lausanne University Hospital and University of Lausanne, Lausanne, Switzerland; 3 Department of Otorhinolaryngology, Lausanne University Hospital and University of Lausanne, Lausanne, Switzerland; 4 Service of Pediatrics, Hospital Riviera-Chablais, Vaud-Valais, Rennaz, Switzerland

**Keywords:** Peritonsillar abscess, Quinsy, Antibiotics, Children

## Abstract

Peritonsillar abscess (PTA) is a common deep neck infection in adolescents and young adults, traditionally treated surgically. This study evaluates the efficacy and safety of conservative management with antibiotics for PTA in pediatric patients. We designed a retrospective observational study at a tertiary care center to analyze the outcome of children under the age of 18 years managed conservatively for PTA from 2004 to 2014. The main outcome was primary treatment failure, defined as the need for surgery or complications within 2 weeks. Secondary outcomes included secondary failure (recurrence or complication more than two weeks after hospital admission) and overall failure (primary or secondary). Of 107 patients, 93 (87%) underwent conservative management with a 6.4% (6/93) primary failure rate requiring surgery and no complications reported. Older age and severe symptoms (e.g., respiratory distress, trismus) correlated with higher failure risk. Secondary failure occurred in 9 patients (9.6%), with recurrences spanning up to 8 years. Overall failure rate was 16.1%, predominantly affecting older children with an adjusted odds ratio per 1-year increase of 1.19 (95% CI 1.03–1.42; *p* = 0.03).

*Conclusions*: Our findings suggest that conservative management of pediatric PTA with antibiotics is a safe and effective approach in selected cases. While most patients respond to antibiotics alone, careful monitoring may be warranted for older children or those with more severe presentations.

**Table:** 

**What is Known:** • *Surgical management is the gold standard for pediatric peritonsillar abscess (PTA).* • *Antibiotic-only treatment is an emerging alternative in selected patients.*
**What is New:** • *This study supports conservative management as safe and effective.* • *Long-term follow-up confirms low failure and complication rates, especially in younger, less symptomatic children.*

**What is Known:**

• *Surgical management is the gold standard for pediatric peritonsillar abscess (PTA).*

• *Antibiotic-only treatment is an emerging alternative in selected patients.*

**What is New:**

• *This study supports conservative management as safe and effective.*

• *Long-term follow-up confirms low failure and complication rates, especially in younger, less symptomatic children.*

## Introduction

Peritonsillar abscess **(**PTA) is the most common deep neck infection in adolescents and young adults. It is defined as a collection of pus located between the tonsillar capsule and the pharyngeal constrictor muscle [1]. PTA typically occurs during peak streptococcal seasons and affects up to 40 cases per 100,000 annually in the Northern hemisphere [2, 3]. Although rare in children under 1 year, 25–30% of cases occur during childhood [4–6].

Clinical presentation includes fever, sore throat, dysphagia, and hallmark features such as trismus and a characteristic “hot potato voice” [7]. On examination, asymmetric swelling of the peritonsillar area, deviation of the uvula, and swelling of the anterior tonsillar pillar are frequently observed [8, 9]. In severe cases, complications such as airway obstruction or spread to adjacent neck spaces can occur. Diagnosis is primarily clinical, though imaging may be used in complex or atypical cases [5, 10].

Surgical intervention, including tonsillectomy or incision and drainage combined with antibiotics, remains the traditional standard of care, with reported success rates around 90% [11–13]. However, interest in conservative management with antibiotics alone has grown [14, 15], with promising outcomes in selected populations [16].

This study evaluates the efficacy and safety of conservative treatment for PTA in children. Our objective was to assess the outcomes of antibiotic-only treatment.

## Material and methods

### Study design

We designed a retrospective observational study at Lausanne University Hospital and recruited pediatric patients diagnosed with PTA between January 1, 2004 and December 31, 2014. With a mainly descriptive focus, our study did not test a specific hypothesis and thus a formal power calculation was not done. We anticipated more than 100 cases of peritonsillar abscess over the study period.

The study was approved by the Commission cantonale d’éthique de la recherche sur l’être humain du Canton de Vaud (CER-VD; 2016–00637; Vaud Cantonal Human Research Ethics Committee), a member of swissethics. The study was conducted in accordance with the Declaration of Helsinki, Good Clinical Practice, and Swiss legal standards. Due to the retrospective nature of the study and the lack of foreseeable risk for the participants, informed consent was not required.

### Population

Children under 18 years of age with a diagnosis of PTA, quinsy or related complications (parapharyngeal abscess, retropharyngeal abscess, Lemierre syndrome, mediastinitis, internal carotid artery pseudoaneurysm) were identified through electronic medical records. Eligibility was based on a retrospective diagnosis of PTA documented in the medical record, typically established clinically by the treating physician. Demographic, clinical, and treatment data were extracted, categorizing patients in two groups: those receiving first-line surgery (surgically treated (ST) group) and those managed conservatively (conservatively treated (CT) group). Conservative treatment consisted of intravenous amoxicillin-clavulanate, transitioned to oral antibiotics upon clinical improvement. Treatment outcomes were then analyzed for children in the CT group.

### Outcomes

Our primary outcome was primary treatment failure defined as the need for surgery or development of complications (para- or retro-pharyngeal abscess, jugular vein septic thrombosis (Lemierre Syndrome), descending necrotizing mediastinitis, internal carotid artery pseudoaneurysm) occurring within 2 weeks of their conservative treatment.

Our secondary outcomes were secondary and overall failures. Secondary failure included recurrence or complications beyond 2 weeks after the start of the conservative treatment and overall failure encompasses both primary and secondary failure.

### Statistical analysis

Demographic and clinical characteristics were compared between groups using Kruskal–Wallis test for continuous variables and Fisher’s exact test for categorical variables. All tests were 2-tailed, and exact *p*-values were calculated. Variables deemed clinically relevant or showing statistical significance (*p*-value < 0.05) were included in a multivariate logistic regression model. Adjusted odds ratios and 95% confidence intervals (CIs) were calculated accordingly. To minimize the risk of overfitting, a multivariate model was computed only if it could include at least one variable per five outcome events. Descriptive statistics were used to describe follow-up. Statistical analyses were computed using Stata software (Stata/IC 11.2 for Mac; StataCorp, Lakeway, TX) and R software, v 4.5.1, and the 2025 R Studio interface (R Studio Team, Boston, MA).

## Results

### Population characteristics

**Fig. 1:**
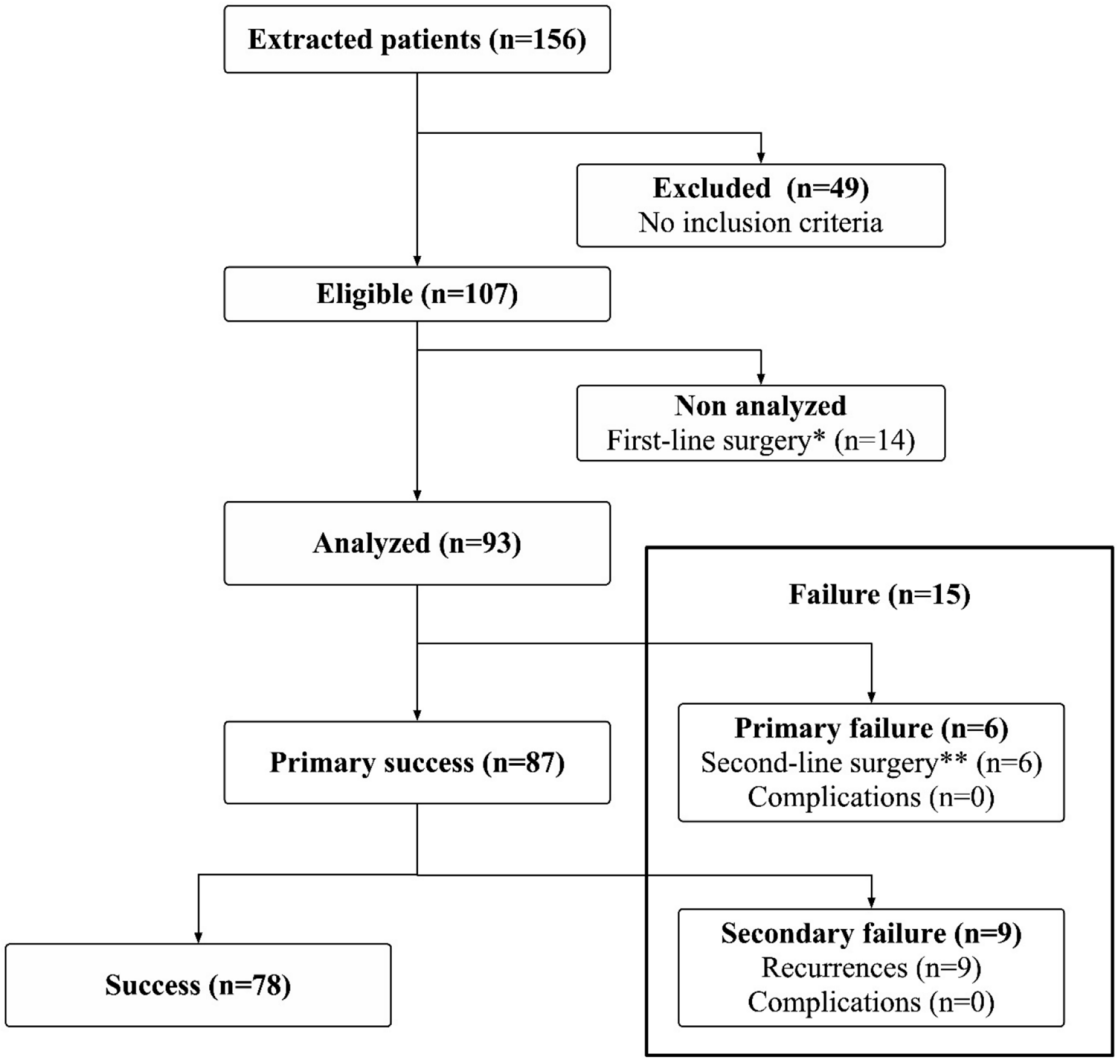
Flowchart of inclusion and group assignment. Legend: *First-line surgery: no attempt at conservative treatment; **Second-line surgery: surgery < 14 days after admission for failure of conservative treatment

From the 156 patients screened, 107 met the inclusion criteria. Among these, 93 patients (87%) were treated conservatively (CT group) while 14 underwent primary surgery (ST group) without an initial attempt at conservative treatment (Fig. 1). Diagnosis was primarily based on clinical evaluation, with computed tomography performed at admission in only 3 of the 107 eligible patients (1 in ST group and 2 in CT group). The only statistically significant difference between groups was age. ST patients were older with a median age of 13.2 years (IQR 7.5–15.8) compared to 12.5 years (IQR 7.1–15.7) for CT patients (*p* = 0.03). Females comprised 59% (55/93) and 43% (6/14) of the CT and ST groups, respectively (*p* = 0.3). Comorbidities were present in 13% (12/93) of the CT group and 7% (1/14) of the ST group (*p* = 1). There was neither a statistically nor a clinically significant difference in clinical presentation between groups (data not shown).

In the CT group, 12 participants had the following underlying health conditions: one had dermatomyositis on methotrexate, one suffered from congenital adrenal hyperplasia, two were diagnosed with asthma, two had mild thalassemia, one was managing type 1 diabetes, one had hepatitis B alongside attention-deficit disorder, one dealt with post-meningitis intracranial hypertension and epilepsy, one faced severe prematurity with numerous concurrent conditions, one had schizencephaly and holoprosencephaly, and one had a diagnosis of hypomelanosis of Ito, significant developmental delay, and obesity.

Only one 11.5-year-old patient of the CT group had a complication of PTA at presentation with a parapharyngeal and left paravertebral abscess with compression of the internal jugular vein. He was treated conservatively with antibiotics due to the proximity of the carotid artery with a favorable outcome.

### Primary failure (CT group)

From the 93 conservatively managed patients, 6 (6.4%) experienced primary failure necessitating surgery but no complications occurred. Patients with primary failure were significantly older with a median age of 15.6 years (IQR 15.3–16.5) vs 11.7 years (IQR 6.9–15.1; *p* = 0.02) and tended to have more severe symptoms such as respiratory distress, trismus, and torticollis (Table 1). All primary-failure patients were older than 13 years of age (range 13.4 to 17.4 years old) and 4 out of 6 had respiratory distress, trismus, and/or torticollis. No multivariate analyses were performed because there were only 6 failure events.

**Table 1:** Determinants of primary failure - univariate analysis

Characteristic, *n* (%)	All subjects, 93 (100)	Primary failure, 6 (6.4)	Primary success, 87 (93.6)	*p*
Gender, *n* female/total (%)	55/93 (59)	5/6 (83)	50/87 (57)	0.4
Age, median years (IQR)	12.5 (7.1–15.7)	15.6 (15.3–16.5)	11.7 (6.9–15.1)	0.02
Comorbidities, *n*/total (%)	12/93 (13)	2/6 (33)	10/87 (11)	0.2
Fever, *n*/total (%)	72/89 (81)	2/5 (40)	70/84 (83)	0.05
Feeding difficulties, *n*/total (%)	83/85 (98)	5/5 (100)	78/79 (98)	1
Hydration difficulties, *n*/total (%)	32/59 (54)	0/1 (0)	32/58 (55)	0.5
Respiratory distress, *n*/total (%)	8/88 (9)	2/5 (40)	6/83 (7)	0.06
Trismus, *n*/total (%)	44/87 (51)	3/4 (75)	41/83 (49)	0.6
Torticollis, *n*/total (%)	12/85 (14)	1/4 (25)	11/81 (14)	0.5

Regarding antibiotic therapy, patients who experienced primary success received treatment for a median duration of 12 days (IQR 11–14), which was comparable to those in the primary failure group, who also had a median treatment duration of 12 days (IQR 5–13) (*p* = 0.47).

The length of intravenous therapy was different between groups with a median of 3 days (IQR 3–4) for the primary success group and 5 days (IQR 4–5) for the primary failure group (*p* = 0.05).

### Secondary and overall failure (CT group)

Secondary failure occurred in nine patients (9.6%) who fulfilled the criteria for at least one PTA recurrence. There were no suppurative complications. At inclusion, secondary-failure patients had a median age of 15.6 years (IQR 15.3–16.5). They presented with their first PTA recurrence at a median time of 16 weeks (IQR 2.9–144, range 15 days to 8 years) after the initial episode and were ultimately all treated with tonsillectomy (one patient after the first recurrence, five patients after their second recurrence, and three patients after their third episode of recurrence).

Overall failure occurred in 15 patients (16.1%). The median age of these 15 patients was 15.4 years (IQR 12.5–16.2) at inclusion, significantly older than the median age of the 78 patients without failure at 11.4 years (IQR 6.5–15; *p* = 0.02) (Table 2). In a multivariate logistic regression analysis, after adjusting for gender and comorbidities, age remained associated with overall failure with an adjusted odds ratio per 1-year increase of 1.19 (95% CI 1.03–1.42; *p* = 0.03) (Table 3).

**Table 2:** Determinants of overall failure - univariate analysis

Characteristic,* n* (%)	All subjects, 93 (100)	Failure, 15 (16.1)	Success, 78 (83.9)	*p*
Gender, n female/total (%)	55/93 (59)	10/15 (67)	33/78 (42)	0.6
Age, median years (IQR)	12.5 (7.1-15.7)	15.4 (12.5-16.2)	11.4 (6.5-15)	0.02
Comorbidities, n/total (%)	12/93 (13)	3/15 (20)	9/78 (12)	0.4

**Table 3:** Determinants of overall failure - multivariate analysis

Characteristic	Contrast	Adjusted odds ratio (95% CI)	*p*
Gender	Female vs Male	1.05 (0.29-3.60)	0.94
Age	1-year increase	1.19 (1.03-1.42)	0.03
Comorbidities	Yes vs No	2.37 (0.44-10.65)	0.27

Twenty-one of the 78 patients (27%) who did not fulfill the criteria for primary or secondary failure had an elective tonsillectomy after a median time of 77.5 days post-diagnosis (IQR 36.5–179.3). When comparing the 21 patients who underwent tonsillectomy to the 57 who did not, there were no significant differences in terms of median age (10.5 years, IQR 6.1–14.9 vs. 11.7 years, IQR 7.8–15.6; *p* = 0.6), presence of comorbidities (5% vs. 13.8%; *p* = 0.4), or gender distribution (70% female vs. 53.4%; *p* = 0.3).

## Discussion

This study underscores the safety and efficacy of conservative management for pediatric PTA. Despite the traditional perception that non-surgical management of PTA is not the standard of care, our findings advocate for its safety and efficacy as a viable alternative to surgical intervention.

With a low primary-failure rate of 6.4% and no complications, our findings align closely with data from the literature. Lamkin et al. reported a failure rate of 4.1% (4/98) in a prospective study of Native American patients treated conservatively for PTA [16]. Recurrence is also a known issue after a first episode of PTA, even after surgery. Rates vary between 6 and 20% following surgical procedures [15, 17]. With 9.6% secondary failure, conservative treatment did not appear to increase this risk in our study. While we focused on the outcome of medical treatment alone, existing data comparing medical and surgical treatment also fail to identify any superiority of one treatment strategy over the other. A comprehensive meta-analysis by Forner et al. [18] encompassing over 33,000 patients (including mixed adult and pediatric cohorts) demonstrated no significant difference in treatment failure rates between medical treatment alone (5.7%) and surgical drainage (5.5%) (odds ratio 1.10; 95% CI 0.53–2.26) [18].

Pain relief and functional recovery constitute other clinical endpoints relevant for treatment choice. The literature shows conflicting results on this topic. The study by Nwe and Singh underscores that incision and drainage yields a more rapid initial improvement in pain and oral intake [19]. Conversely, Battaglia et al. reported that total pain duration and opioid consumption were actually lower in the “antibiotic only” group. This may suggest that while immediate symptom relief may be superior with drainage, the overall recovery can be just as favorable with conservative management in selected cases [15].

Forner et al. underscore in their meta-analysis that they could not differentiate clinical scenarios that justify either therapeutic option. They emphasize that carefully selecting patients who are suitable for medical management is essential to making conservative treatment successful [18]. Despite the high success rate, we found that a certain subpopulation of older and more symptomatic patients had a higher risk of failure. Although trismus, torticollis, and respiratory distress did not reach statistical significance as predictors of failure, they remain highly relevant in practice. Other research has similarly identified patient characteristics associated with outcomes in pediatric cases. For instance, Kim et al. found that younger age (under 7.5 years), fewer episodes of acute tonsillitis, and smaller abscess size were predictors of successful conservative treatment [20]. In addition, Battaglia et al. highlighted that children who experienced recurrences were more likely to have comorbidities and larger abscesses requiring surgical intervention [15].

The variability of treatment duration is a trend consistent with contemporary clinical practice. Notably, a survey by Wu et al. found that outpatient antibiotic courses for PTA ranged from 5 to 14 days, most often 10 days, highlighting the lack of standardization and confirming that the durations observed in our study are well within current norms [21]. Patients who experienced primary failure required a longer duration of intravenous treatment, likely due to their slower initial clinical improvement. Nevertheless, this did not result in a significant difference in the overall length of antibiotic therapy. Although the extended follow-up in our study is a notable strength, offering comprehensive insight into recurrence rates and long-term efficacy, it is important to acknowledge several limitations. First, patients in the ST groups were significantly older than those in the CT group. Since the risk of primary and overall failure increased with age, this age disparity may have led to an underestimation of failure rates. Additionally, while the diagnosis was primarily based on clinical evaluation (with only 2 computed tomography scans performed in the CT group), this approach may have introduced selection bias. Misclassification is possible, but it typically involves differentiating peritonsillar abscess from other causes of tonsillar asymmetry, such as constitutional asymmetry or intratonsillar abscess. However, the presence of anterior pillar bulging remains a key diagnostic criterion that usually distinguishes peritonsillar abscess from these other entities. Therefore, we believe that the risk of misclassification remained low and that clinical diagnosis remains an accepted approach for diagnosing PTA with radiological imaging only considered essential in complicated cases [22]. Notably, radiological imaging was specified as an inclusion criterion in only two of twelve studies in Forner’s systematic review [18]. It is also worth noting that radiological assessments have drawbacks. For example, Eliason et al. found that up to 30.4% of CT scans interpreted as showing abscesses did not reveal purulence at intervention [23]. Furthermore, the small number of failure events and the retrospective nature of the study limit the strength of our conclusions regarding predictors of primary treatment failure. Lastly, IV to oral transition practices and total antibiotic therapy duration were not standardized. The optimal management strategy, such as the best duration for intravenous antibiotics before transitioning to oral treatment, remains an open question for future research [21].

## Conclusion

Our findings suggest that conservative management of pediatric PTA with antibiotics is a safe and effective approach in selected cases. While most patients respond to antibiotics alone, careful monitoring may be warranted for older children or those with more severe presentations. While our results are encouraging, they must be interpreted in the context of their limitations. In light of our findings, future studies could develop and validate clinical decision-making algorithms to assist in identifying pediatric patients most likely to benefit from conservative management. Such algorithms might incorporate variables including age; symptom severity (notably the presence of trismus, respiratory distress, or torticollis); comorbidities; abscess size if available; and early response to intravenous antibiotics. Establishing clear patient selection criteria within prospective studies would facilitate more individualized treatment strategies and help standardize care, ultimately optimizing outcomes for children with peritonsillar abscess.

## Data Availability

No datasets were generated or analysed during the current study.

## Abbreviations

CI: Confidence interval
CT: Conservatively treated
IQR: Interquartile range
IV: Intravenous
PTA: Peritonsillar abscess
ST: Surgically treated
